# Frontocortical activity and emotional experience in the context of daily life events

**DOI:** 10.1093/scan/nsaf102

**Published:** 2025-10-06

**Authors:** Nayoung Kim, Hakin Kim, Chae-eun Chung, Junhyun Park, M Justin Kim, Juyoen Hur

**Affiliations:** Department of Psychology, Yonsei University, Seoul 03722, Republic of Korea; Department of Psychology, Yonsei University, Seoul 03722, Republic of Korea; Department of Psychology, Yonsei University, Seoul 03722, Republic of Korea; Department of Psychology, Yonsei University, Seoul 03722, Republic of Korea; Department of Psychology, Sungkyunkwan University, Seoul 03063, Republic of Korea; Center for Neuroscience Imaging Research, Institute for Basic Science, Suwon 16419, Republic of Korea; Department of Psychology, Yonsei University, Seoul 03722, Republic of Korea

**Keywords:** momentary emotional experience, positive affect, frontocortical brain

## Abstract

Understanding the neurobiological basis of emotional experience in the context of daily life events is crucial for elucidating the mechanisms of emotion and emotion regulation, as well as for developing novel interventions for emotion-related disorders. Frontocortical brain regions, including the dorsolateral prefrontal cortex and mid-cingulate cortex, are thought to contribute to emotional processing and regulation and have been proposed as potential biomarkers of individual emotional well-being. However, how these regions relate to emotional experience across daily event contexts remains poorly understood. By integrating fMRI and ecological momentary assessment, the present study investigated whether, and how, frontocortical activity measured in the laboratory is associated with positive and negative emotional experience in the presence and absence of relevant daily events. Multilevel analyses revealed that individual differences in frontocortical activity were significantly associated with positive, but not negative, emotional experience. Specifically, individuals with heightened frontocortical activity exhibited significantly elevated baseline positive mood in the absence of positive events, compared to those with low frontocortical activity. These findings offer novel insights into the neural mechanisms underlying emotional dynamics and well-being in real-world settings.

## Introduction

Our daily lives are filled with a mixture of positive and negative events, eliciting a range of emotional experiences. As noted by Davidson (1998), the significant variability in emotional response patterns is a striking aspect of human emotion. Understanding the neurobiological underpinnings of individuals’ emotional experience in the context of daily life events is crucial for comprehending the fundamental mechanisms of emotion and emotion regulation, and for developing novel interventions to address emotion-related psychopathology.

Frontocortical brain regions, including the dorsolateral prefrontal cortex (dlPFC) and mid-cingulate cortex (MCC), play a central role in emotional processing and regulation. The dlPFC is critically involved not only in top-down cognitive control but also in the implementation of emotion regulation strategies, such as reappraisal (Nelson et al. 2015, Ma et al. 2017, Kroes et al. 2019). It has also been posited to function as a shared resource for managing both cognitive and emotional demands, thereby facilitating interactions between these systems (Pessoa 2015). Consistent with this, affective dysfunction is often accompanied by deficits in cognitive control (Eysenck et al. 2007), and failure to engage the dlPFC during attentional control or in affectively challenging contexts (e.g. receiving criticism) has been linked to increased vulnerability to mood and anxiety disorders (Hooley et al. 2005, Bishop 2009). Similarly, the MCC has been identified as a hub connecting emotional and executive systems (Pessoa 2008, Shackman et al. 2011). The MCC is considered to be involved in detecting salient emotional stimuli (Seeley et al. 2007, Touroutoglou et al. 2012) and in resolving conflicts to guide adaptive responses (Shackman et al. 2011, Sheth et al. 2012). Together, these regions interact dynamically to support flexible modulation of emotional experiences in the context of diverse life events (Ochsner and Gross 2005, Bo et al. 2024). Recent findings further indicate that these frontocortical regions are spontaneously engaged in minimizing distress and inhibiting threat-induced behaviours (Fitzgerald et al. 2020, Wang et al. 2024), underscoring their role in regulating negative affect.

Growing evidence also points to a link between frontocortical activity and positive emotional experience. For example, Park et al. (2023) found that individuals with greater dlPFC reactivity to happy faces reported higher life satisfaction and overall positivity. Similarly, sustained dlPFC activation in response to positive images has been associated with greater eudaimonic well-being, including self-acceptance, positive relationships, and a sense of purpose (Heller et al. 2013). Moreover, dlPFC activity during reward processing correlates with stronger emotional responses to positive stimuli (Heller et al. 2015), and MCC activity predicts elevated positive emotion following hedonic activities such as gaming (Hickey et al. 2010). Both the dlPFC (Engen and Singer 2015, Doré et al. 2017) and the MCC (Johnston et al. 2011, Greening et al. 2014) have also been shown to be recruited during efforts to actively upregulate positive affect. Together, these findings suggest that frontocortical function may also support the enhancement of positive emotional experiences.

However, with a few exceptions (e.g. Heller et al. 2015), most prior studies (e.g. Hickey et al. 2010, Park et al. 2023) have assessed positive emotions as trait affect or general mood at a single time point, limiting insight into how frontocortical activity relates to momentary emotional fluctuations. In addition, most related neuroimaging research has relied on passive viewing or reward tasks, leaving the relationship of positive emotional experience and frontocortical activity during cognitive control unclear. Also, much of the existing work (e.g. Mak et al. 2009, Heller et al. 2013, Fitzgerald et al. 2020, Park et al. 2023) is based on affect measured through retrospective self-reports or in controlled experimental settings, which compromises ecological validity (but see Heller et al. 2015, Grosse Rueschkamp et al. 2019, Hur et al. 2022). Ecological momentary assessment (EMA) addresses this limitation by capturing real-time emotional experiences in naturalistic environments, offering a unique window into daily affective dynamics.

By integrating fMRI and EMA techniques, the present study examined how frontocortical activity during a pure cognitive control task (i.e. the Stroop task), a potential neural marker of emotional well-being, relates to positive and negative emotional experiences in daily life, both in the presence and absence of relevant life events. We hypothesized that individuals with greater frontocortical activity would exhibit lower negative emotional reactivity, reflected in a smaller increase in negative affect in response to negative events. We also predicted that heightened frontocortical activity would be associated with enhanced positive emotion, either as increased positive affect in response to positive events or as elevated baseline positive mood in the absence of such events. This study aims to bridge the gap between neural mechanisms and real-world emotional experiences, shedding light on how frontocortical function relates to daily emotional life.

## Material and Methods

### Overview

This study was part of an ongoing prospective longitudinal study investigating etiological mechanisms underlying risk for depression and anxiety disorders. All participants were Korean young adults under 25 years old residing in the Seoul metropolitan area, with consistent access to a smartphone for EMA (see online supplementary material for details). Participants reported no history of lifetime neurological disorders, MRI contraindications, current internalizing disorders (e.g. major depressive disorder, generalized anxiety disorder, panic disorder, social anxiety disorder), past or current alcohol/substance abuse, suicidal ideation, psychiatric treatment, or psychiatric medicine use (see online supplementary material). During the baseline laboratory session, participants provided written informed consent and were familiarized with the EMA protocol. Starting the following day, participants completed up to five EMA surveys per day for 14 days and underwent a neuroimaging assessment. All procedures were approved by the Institutional Review Board (IRB) of Yonsei University, Seoul, South Korea.

### Participants

A total of 118 participants completed both the EMA protocol and the fMRI assessment. Out of these, nine participants were excluded from EMA analyses due to a low response rate (less than 50%). From the remaining 109 participants with usable EMA data, 25 were further excluded from fMRI analyses of the colour-word Stroop task for the following reasons: coil issues (*n *= 8), technical issues (*n *= 3), anatomical brain lesions (*n *= 2), low task compliance (*n *= 2), excessive movement in the scanner (*n *= 7), and fieldmap errors (*n *= 3). This yielded a final sample of 84 participants (77.7% female; *M *= 22.3 years, *SD *= 2.2). All procedures were approved by the Institutional Review Board (IRB) of Yonsei University, Seoul, South Korea. The sample partially overlaps with that used in our prior work examining the neurobiological correlates of neuroticism (Kim et al. 2025).

### EMA procedures

#### Protocol

**Figure 1.**
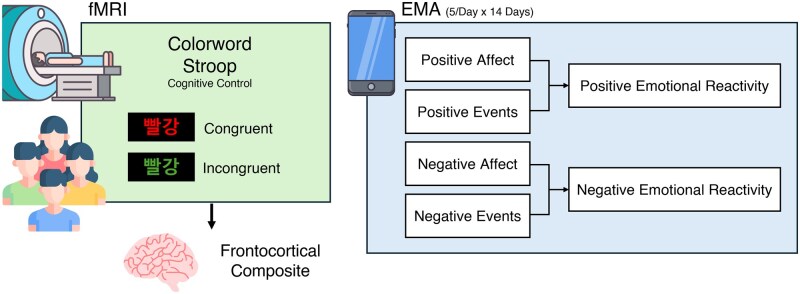
Study overview. Participants completed a colour-word Stroop task during fMRI and a 14-day ecological momentary assessment (EMA) protocol. ROIs were defined based on significant group-level activation (FDR *q* < .05, whole-brain corrected) within anatomically relevant regions identified in prior work. Based on our hypotheses, analyses focused on two frontocortical ROIs: dlPFC and MCC. To reduce comparisons and enhance power, we created a composite score. EMA captured momentary fluctuations in positive and negative affect in the presence and absence of daily events, with up to five surveys per day, yielding 4466 usable assessments. HLMs probed associations between the composite neural metric and emotional experience. The example trial illustrates the Stroop word “red,” presented in Korean. dlPFC, dorsolateral prefrontal cortex; MCC, midcingulate cortex; ROIs, regions of interest; FDR, false discovery rate; HLM, hierarchical linear models.

Participants completed up to five EMA surveys per day for 14 days to capture fluctuations in momentary affect and experiences of positive and negative events (Fig. 1). Over a span of 14 days, five prompt messages were delivered pseudo-randomly within a designated 12-hour period each day via smartphone text message. Participants had the option to receive their first prompt between 7:00 a.m. and 12:00 p.m., based on personal preference. Each prompt included a link to a secure online survey. Participants were instructed to complete the survey within 15 minutes and to avoid responding in unsafe or inconvenient situations (e.g. while driving). On average, participants spent approximately three minutes completing each survey. Several strategies were employed to enhance compliance, including offering monetary bonuses for higher response rates, and providing participants with updates on their overall response rate on the second and seventh days of the study.

#### EMA survey and data reduction

Participants rated their current positive affect (PA: enthusiastic, joyful, cheerful, calm, content, relaxed) and negative affect (NA: nervous, worried, afraid, sad, hopeless, downhearted, irritated, angry, tired, lonely) on a 5-point scale ranging from 1 (not at all) to 5 (extremely). Data reduction was conducted through exploratory factor analysis (EFA) utilizing the *nFactors* (Raiche et al. 2020) and *psych* package (Revelle 2020) in R. The EFA revealed a two-factor solution at the between-person level, with factor 1 comprising all six PA items, and factor 2 encompassing all ten NA items (Table S1, see online supplementary material for a colour version of this table). Composite measures of positive and negative affect were calculated by averaging the respective items. Both measures demonstrated high internal consistency (*αs* > .86). The occurrence of positive or negative events was assessed using separate binary items: ‘Did you experience a positive event since the previous prompt?’ and ‘Did you experience a negative event since the previous prompt?’, with responses coded as ‘0’ for no and ‘1’ for yes. The final sample demonstrated acceptable EMA compliance (*M *= 76.0%, *SD *= 10.41%, minimum = 51.43%, total assessments = 4466).

### fMRI task

The colour-word Stroop task was employed to assess frontocortical activation levels during a pure cognitive control task (Figure S1, see online supplementary material for a colour version of this figure). This task has shown moderate test-retest reliability of fMRI BOLD signal change, and similar tasks have been widely employed to assess frontocortical function as a biomarker in large-scale studies, including the Adolescent Brain Cognitive Development (ABCD) Study, the Human Connectome Project (HCP), and the UK Biobank (Sheu et al. 2012, Glasser et al. 2013, Fawns-Ritchie and Deary 2020, Smolker et al. 2022). During this task, each trial displayed a target word at the centre of the screen, with four identifier words presented in a single row at the bottom. These words were the Korean terms for ‘blue’, ‘yellow’, ‘green’, and ‘red’. In the congruent condition, the colour of the target word matched its semantic meaning (e.g. the word ‘red’ displayed in red), whereas in the incongruent condition, there was a mismatch between the colour and the meaning of the target word (e.g. the word ‘red’ displayed in green). The identifier words were displayed in white.

Participants were asked to report the colour of the target word by pressing one of four buttons corresponding to the identifier words, as quickly and accurately as possible. The task included two runs, each composed of 12 blocks (6 congruent and 6 incongruent blocks) The order of the blocks was counterbalanced across participants. Each block lasted 30 s, containing 12 trials with randomized presentation. Trials featured a stimulus duration of 2 s and an interstimulus interval of 0.5 s. Participants were allowed a rest period of 19.5 s after every three blocks, totalling three rest periods per run. Participants demonstrating inadequate performance (accuracy < 2 *SD* below the mean for both scans) were excluded from analysis.

### MRI data acquisition

MRI data were acquired using a Siemens MAGNETOM Vida 3-tesla scanner (32-channel head coil). T1-weighted anatomical images were acquired using a magnetization-prepared rapid-acquisition gradient echo (MPRAGE) sequence (TR = 2300 ms, TE = 2.26 ms flip angle = 8°, field of view = 256 mm, matrix = 256 × 256, voxel size 1 × 1 × 1 mm^3^). For enhanced resolution, a multiband sequence was employed to collect echo-planar imaging (EPI) volumes (multiband acceleration = 3, TR = 1500 ms, TE = 30 ms, flip angle = 80°, field of view = 220 mm, matrix = 110 × 110, 69 transversal slices, interleaved slice acquisition, voxel size 2 × 2 × 2 mm^3^). Each run of the colour-word Stroop task comprised 283 volumes. Additionally, for distortion correction, 10 EPI images with reversed phase encoding direction (i.e. posterior to anterior) were acquired along with double-echo gradient EPI images (TR = 672 ms, TE 1 = 4.92 ms, TE 2 = 7.38 ms, flip angle = 60°, field of view = 220 mm, matrix = 110 × 110, 69 transversal slices, voxel size 2 × 2 × 2 mm^3^) to generate two magnitude images and a single phase difference image.

### MRI data processing

Image data were preprocessed using *fMRIPrep 22.1.1* (Esteban et al. 2019), a tool built on *Nipype 1.8.5* (Gorgolewski et al. 2011; see online supplementary material for details).

#### Functional data

A reference volume and its skull-stripped version were generated using a custom methodology in *fMRIPrep*. Head-motion parameters with respect to the BOLD reference (including transformation matrices and six corresponding rotation and translation parameters) were estimated using *mcflirt* (FSL, Jenkinson et al. 2012) prior to any spatiotemporal filtering. The estimated fieldmap was aligned to the target EPI reference run using rigid registration. Field coefficients were then mapped onto the reference EPI using the calculated transform. BOLD runs were slice-time corrected using *3dTshift* from AFNI (Cox and Hyde 1997). The BOLD reference was then co-registered to the T1w reference using *bbregister* (FreeSurfer), which implements boundary-based registration (Greve and Fischl 2009), configured with six degrees of freedom. The BOLD time series were resampled into standard space, producing a preprocessed BOLD run in MNI152NLin6Asym space. Motion artifacts were automatically removed using independent component analysis (ICA-AROMA, Pruim et al. 2015), following the removal of non-steady state volumes and spatial smoothing with an isotropic Gaussian kernel of 6 mm FWHM. Corresponding ‘non-aggressively’ denoised runs were produced after such smoothing.

### fMRI modelling and data reduction

The preprocessed images were analysed using *SPM12* (https://www.fil.ion.ucl.ac.uk/spm) and custom MATLAB scripts. A temporal high-pass filter with a cutoff frequency of 128 s was applied. Regressors were convolved with a canonical HRF and its temporal derivative. Volumes with non-steady state outliers were regressed out. The general linear model (GLM) included regressors for congruent and incongruent trials for both runs, employed in a block design. Analyses focused on frontocortical activity during incongruent trials (compared to congruent trials), consistent with prior work (Leung et al. 2000, Huang et al. 2020). Individual contrast images (incongruent vs. congruent) were generated and submitted to a second-level random effects model, followed by a one-sample *t*-test. Significance was assessed using false discovery rate (FDR) *q* < .05, whole-brain corrected.

#### Brain metrics

We functionally defined regions of interest (ROIs) at the group level based on significant task effects observed in the whole brain analysis [Table S2 (see online supplementary material for a colour version of this table) and Figure S2 (see online supplementary material for a colour version of this figure); FDR *q* < .05, whole-brain corrected] within the bilateral dlPFC and MCC selected on the basis of prior large-scale studies and meta-analyses (Hung et al. 2018, Huang et al. 2020). To define the bilateral dlPFC as an ROI, we obtained a meta-analytic mask from Neurosynth (Yarkoni et al. 2011) the search term ‘dlPFC’. This mask was multiplied with the anatomical automatic labelling (AAL) masks of the precentral gyrus, superior frontal gyrus, middle frontal gyrus, and inferior frontal gyrus to ensure anatomical specificity. Non-brain voxels were excluded using an MNI152 brain mask. The refined bilateral dlPFC mask was then overlaid onto the task effect map [Figure S2 (see online supplementary material for a colour version of this figure); FDR *q* < .05, whole-brain corrected] and 6 mm spheres were centred on the left and right local maxima (see Table S3 for coordinates, see online supplementary material for a colour version of this table). For the MCC, we constructed an ROI using a meta-analytic map related to ‘cognitive control’ obtained from Neurosynth. This mask was multiplied with the midcingulate gyrus AAL mask to enhance specificity and superimposed onto the task effect map to delineate 6 mm spheres centred at the peak coordinates within the MCC (see Table S3 for coordinates, see online supplementary material for a colour version of this table). For hypothesis testing, the two ROIs, the bilateral dlPFC and MCC ROIs, were combined to form a single frontocortical ROI. Contrast values (inc vs. con) were extracted from the frontocortical ROI to quantify frontocortical activity during the colour-word Stroop task.

### Hypothesis testing strategy

We integrated the fMRI and EMA data streams through hierarchical linear models (HLMs), a form of mixed-effects modelling that accounts for the nested structure of repeated measures within individuals, to examine the relationship between frontocortical brain activity and both daily positive and negative emotional experience. HLMs were computed using R (version 4.2.1) with the *lme4* (Bates et al. 2015) and *lmerTest* (Kuznetsova et al. 2017) packages.

First, to test the link between frontocortical activity and positive emotional experience in the presence and absence of positive events, we nested the EMA-derived time series of positive affect (continuous) and exposure to positive events (binary) within participants (Level 1 variables). Intercepts were free to vary across participants. Brain metrics (frontocortical activity) were grand-mean centred and served as a continuous Level 2 predictors. The equations below outline the basic structure of the HLM in standard notation (Raudenbush and Bryk 2002). At the first level, positive affect during EMA at time *t* for individual *i* was modelled as a function of exposure to positive events. The absence of exposure to positive events served as the reference category (baseline):


(1a) $${\mathrm{positive}\;\mathrm{affect}}_{ti}=\pi_{0i}+\pi_{1i}(\mathrm{positive}\;\mathrm{event})+e_{ti}$$

At the second level of the HLM, the relationship between positive events and positive affect was modelled as a function of individual differences in frontocortical activity:


(1b) $$\pi_{0i}=\beta_{00}+\beta_{01}({\mathrm{frontocortical}\;\mathrm{activity}}_{i})+r_{0i}$$

(1c) $$\pi_{1i}=\beta_{10}+\beta_{11}({\mathrm{frontocortical}\;\mathrm{activity}}_{i})+r_{1i}$$

Conceptually, individual differences in positive emotional experience in the presence vs. absence of positive events were estimated using a binary reference function that indicated the self-reported presence or absence of positive event exposure at each EMA (Equation (1a)). In this model, the slope $\pi_{1i}$ in Equation (1a) reflects the extent to which an individual’s positive affect changes when a positive event occurs. The interaction term (i.e. frontocortical activity × positive event) captures how frontocortical activity is associated with positive affect in the presence vs. absence of positive events (Table 1).

**Table 1.** MLM results.

	Positive affect				Negative affect		
Factor	*t*	*β*	SE	Factor	*t*	*β*	SE
Frontocortical	0.77	0.13	0.17	Frontocortical	−0.13	−0.02	0.12
Positive event (vs. absent)	19.96**	0.60	0.03	Negative event (vs. absent)	11.85**	0.48	0.04
Frontocortical × positive event	−2.87*	−0.23	0.08	Frontocortical × negative event	−0.52	−0.05	0.10

Relations between brain metrics and emotional experience in the presence and absence of relevant daily events.

* *P* < .01;

** *P* < .001.

The relationship between frontocortical activity and negative emotional experience in the presence and absence of negative events was modelled using parallel methods. At the first level, negative affect during EMA at time *t* for individual *i* was modelled as a function of exposure to negative events. The absence of exposure to negative events served as the reference category (baseline):


(2a) $${\mathrm{negative}\;\mathrm{affect}}_{ti}=\pi_{0i}+\pi_{1i}(\mathrm{negative}\;\mathrm{event})+e_{ti}$$

At the second level of the HLM, the relationship between negative events and negative affect was modelled as a function of individual differences in frontocortical activity:


(2b) $$\pi_{0i}=\beta_{00}+\beta_{01}({\mathrm{frontocortical}\;\mathrm{activity}}_{i})+r_{0i}$$

(2c) $$\pi_{1i}=\beta_{10}+\beta_{11}({\mathrm{frontocortical}\;\mathrm{activity}}_{i})+r_{1i}$$

Individual differences in negative emotional experience in the presence vs. absence of negative events were estimated using a binary reference function indicating the presence or absence of negative event exposure at each EMA (Equation (2a)). In this model, the slope $\pi_{1i}$ in Equation (2a) reflects the extent to which an individual’s negative affect changes when a negative event occurs. The interaction term (e.g. frontocortical activity x negative event) captures how frontocortical activity is associated with negative affect in the presence vs. absence of negative events (Table 1).

The Šidák procedure was used to determine corrected two-tailed significance thresholds for multiple tests conducted. This method adjusts the significance level for each test to maintain the overall probability of making at least one Type 1 error within a family of tests at the desired alpha level and is known to be less conservative than the Bonferroni correction (Abdi 2007). Control analyses were conducted on significant results, controlling for age, sex, and the frequency of exposure to positive (or negative) events. Although our primary focus was on the results from the frontocortical composite, follow-up analyses were conducted to examine the bilateral dlPFC and MCC as separate ROIs, in order to clarify whether they exhibit differential associations with daily experiences of positive and negative affect.

## Results

### Stroop task results: behavioural performance and brain activation

#### Behavioural results

Paired *t*-tests revealed that incongruent trials were associated with significantly longer reaction times (*t*(83) = 19.14, *P*  <  .001) and higher error rates (*t*(83) = 6.11, *P * <  .001) compared to congruent trials, confirming the Stroop interference effect (Table S4, see online supplementary material for a colour version of this table).

#### fMRI results

As expected, the incongruent vs. congruent contrast revealed significant activation in regions implicated in cognitive control, including the MCC and dlPFC [FDR *q* < .05, whole-brain corrected; Table S2 (see online supplementary material for a colour version of this table) and Figure S2 (see online supplementary material for a colour version of this figure)].

Also consistent with expectations, the experience of positive events was associated with an increase in momentary positive affect (*β*  =  0.60, *P* < .001), whereas the experience of negative events was associated with an increase in momentary negative affect (*β*  =  0.48, *P* < .001). The main effect of frontocortical activity on either positive or negative affect was not significant.

**Figure 2.**
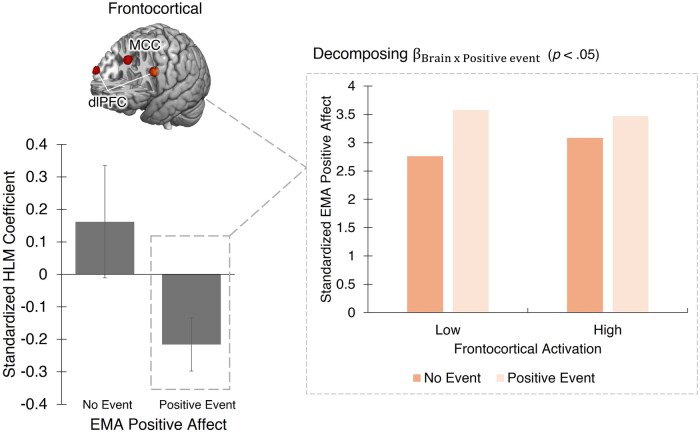
Relationship between frontocortical activity and positive emotional experience in the presence and absence of positive daily events. For illustration, significant interactions are plotted at ±1 SD from the mean of frontocortical activity.

Next, we examined the relationship between frontocortical activity and positive and negative emotional experience in the presence and absence of relevant events in daily life. We found that individual differences in frontocortical activity were significantly associated with positive, but not negative, emotional experience in the real world (*β* = −0.23, *P* = .005; Fig. 2 and Table 1). Specifically, individuals with heightened frontocortical activity exhibited significantly elevated baseline positive mood in the absence of positive events, compared to those with low frontocortical activity. These results remained consistent after controlling for age, sex, and the frequency of exposure to positive events (Table S5, see online supplementary material for a colour version of this table) and even after adjustment for multiple comparisons (Šidák $\alpha_{\mathrm{critical}}$= .025). In contrast, individual differences in frontocortical activity were not associated with negative emotional experience in the presence or absence of negative events (*β* = −0.05, *P* = .60; Table 1).

When the bilateral dlPFC and MCC were considered separately, results were consistent with those from the frontocortical composite: individual differences in both the bilateral dlPFC and MCC were significantly associated with positive, but not negative, emotional experience in the real world (*P*s < .05). Both associations remained significant after applying a Šidák correction for multiple comparisons (Šidák $\alpha_{\mathrm{critical}}$= .025), each showing a pattern similar to that observed for the frontocortical composite (Tables S6 and S7, see online supplementary material for a colour version of these tables). Neither the bilateral dlPFC nor the MCC were significantly associated with momentary negative affect in the presence or absence of negative events (Tables S6 and S7, see online supplementary material for a colour version of these tables).

## Discussion

The present study utilized a combination of fMRI and EMA to investigate how individual differences in frontocortical activity, measured during a laboratory-based cognitive control task (i.e. the Stroop task), relate to emotional experiences in daily life. By examining both positive and negative emotional experiences, we found that individual differences in frontocortical activity were significantly associated with positive, but not with negative, emotional experiences, particularly in the absence of relevant daily events. These findings offer novel insights into the neural mechanisms underlying real-world emotional dynamics and lay the groundwork for identifying new treatment targets for emotion-related disorders.

Our findings clarify the manner in which frontocortical activity were related to positive emotional experience across different daily life event contexts. Specifically, individuals with heightened frontocortical activity exhibited significantly greater baseline positive mood in the absence of positive events, compared to those with lower frontocortical activity. These findings suggest that frontocortical activity associated with cognitive control may play a role in stabilizing momentary positive affect when positive events are absent (Kuppens et al. 2007, Daly et al. 2014), rather than amplifying positive emotion in response to such events. This aligns with previous studies linking greater frontocortical activity to enhanced psychological well-being (Heller et al. 2013, King 2019, Park et al. 2023) and positivity (Habel et al. 2005). Clinical research further supports this view, showing that individuals with anhedonia, defined as the inability to maintain stable positive affect (Tomarken and Keener 1998, Liu et al. 2011, Horner et al. 2014), often exhibit reduced or dysregulated activity in frontocortical regions such as the dlPFC and anterior MCC (Greening et al. 2014, Henderson et al. 2014, Fan et al. 2024). Our results extend these findings by demonstrating that heightened frontocortical activity may foster everyday emotional stability, particularly in the absence of overtly positive environmental cues.

Contrary to our expectations, frontocortical activity was not significantly associated with negative emotional experience in the presence or absence of relevant events. Two possibilities may account for this finding. First, the lack of association may reflect our sample characteristics. As is typical in healthy samples, participants in our study reported low average levels of negative affect with limited variability (*M *= 1.60, *SD *= 0.61). The frequency of negative events (*M *= 0.12, *SD *= 0.14) was also low. This restricted range may have produced a floor effect, potentially obscuring a true relationship between frontocortical activity and negative emotional experience (von Klipstein et al. 2023).

Second, it is possible that frontocortical activity does not meaningfully modulate negative affect on a day-to-day basis. Many prior studies demonstrating such associations have relied on laboratory-based emotion induction paradigms (e.g. Goldin et al. 2008, Balderston et al. 2017), which may not reflect the complexity of negative emotional experiences in real-world settings (Um et al. 2023). The relationship between frontocortical activity and real-world negative affect may be more nuanced and context-dependent (Komulainen et al. 2014, Brockman et al. 2017). For example, increased frontocortical engagement may be associated with decreased negative affect in some situations but with increased negative affect in others (Kalisch and Gerlicher 2014, Sokołowski et al. 2022), depending on individual goals, regulatory strategies, or situational demands. This complexity is also echoed in a recent EMA–fMRI fusion study (Hur et al. 2022), which found that greater frontocortical activity during a threat anticipation task was associated with reduced negative emotional reactivity. Notably, unlike that study, we used a pure cognitive control task (i.e. Stroop task), which may better capture general, context-independent top-down control capacity. However, the opposite may also be true—that context-specific tasks (e.g. a cognitive control task with emotional distractors) are more sensitive to detect a meaningful relationship between the two. This methodological distinction may help explain the divergent findings and highlights the need for further research integrating EMA and fMRI to clarify these relationships.

Our findings suggest that individual differences in frontocortical activity are closely associated with the stability of momentary positive affect in the absence of positive events in daily life. Increasing evidence highlights the importance of maintaining positive affect, not just reducing negative affect, in treating and preventing depression and stress-related disorders (Santos et al. 2013, Silton et al. 2020). These results support the potential value of cognitive training or neurofeedback interventions aimed at enhancing frontocortical function to promote emotional well-being. Additionally, our findings point to the utility of frontocortical activity as a candidate biomarker for emotional functioning in real-world contexts—one that could help identify individuals at risk and guide more personalized treatment approaches.

Despite these novel insights, several limitations warrant consideration. First, although our results remained consistent after controlling for gender, our sample was predominantly female (77.7%), which may limit the generalizability of our findings. Future research is needed to replicate these findings in more gender-balanced samples. Second, although we observed significant associations between a laboratory-based neural marker and real-world emotional experience, causality cannot be inferred. Future longitudinal or intervention studies are needed to determine whether frontocortical activity predicts stable patterns of positive affect. Third, frontocortical regions do not operate in isolation; rather, they function as part of larger networks. Future studies should examine how frontocortical areas interact with reward-related (e.g. striatum) and threat-related (e.g. amygdala) systems to support affective dynamics. Fourth, the link between fMRI-derived neural markers and EMA-measured affect may be shaped by contextual and psychological factors (e.g. emotion regulation strategies, cognitive load). Incorporating richer contextual and behavioural data would allow for more nuanced interpretations of these associations. Finally, as this study focused on a non-clinical sample, future research should examine whether these findings generalize to clinical populations and contribute to understanding the mechanisms underlying mood and anxiety disorders.

This study is among the few to integrate fMRI and EMA data to demonstrate that individual differences in frontocortical activity are associated with the stability of momentary positive affect across relevant daily life events. The present findings offer novel insights into the neurobiological substrates of emotional well-being in real-life settings.

## Supplementary Material

nsaf102_Supplementary_Data

## Data Availability

The processed data and analysis scripts for this study are publicly available on the Open Science Framework (OSF) website at https://osf.io/4235b/.
