# Supplementary material for: Frontocortical activity and emotional experience in the context of daily life events
Source: Soc Cogn Affect Neurosci. 2025 Oct 6;20(1):nsaf102. doi: 10.1093/scan/nsaf102 (PMC12602155; doi:10.1093/scan/nsaf102)
Supplement: nsaf102_Supplementary_Data [file nsaf102_supplementary_data.docx]

**Frontocortical activity and emotional experience in the context of daily life events**

Nayoung Kim^1^

Hakin Kim^1^

Chae-eun Chung^1^

Junhyun Park^1^

M. Justin Kim^2, 3^

Juyoen Hur^1^

^1^ Department of Psychology, Yonsei University, Seoul 03722, Republic of Korea

^2^ Department of Psychology, Sungkyunkwan University, Seoul 03063, Republic of Korea

^3^ Center for Neuroscience Imaging Research, Institute for Basic Science, Suwon 16419, Republic of Korea

**Abbreviated Title:** Frontocortical activity and daily emotion

**Key Words:** momentary emotional experience, positive affect, frontocortical brain

**Address Correspondence to:**

Juyoen Hur ([jhur1@yonsei.ac.kr](mailto:jhur1@yonsei.ac.kr))

Address: Yonsei University, Widang Hall #509-1, 50, Yonsei-ro, Seodaemun-gu, Seoul, 03722, Republic of Korea

Phone: (82-2) 2123-2440 / Fax: (82-2) 365-4354

**Table of Contents**

**Supplementary Method**

Additional information about the current study p. 3

Measurement of trait negative emotionality p. 4

Figure S1. Color-word Stroop paradigm p. 4

MRI data processing p. 5

**Supplementary Results**

Table S1. Results of the exploratory factor analysis on EMA affect items p. 6

Figure S2. fMRI task effects p. 7

Table S2. Descriptive statistics for clusters showing significant activation in the Incongruent

> Congruent contrast p. 8

Table S3. Descriptive statistics for local maxima within the bilateral dlPFC and MCC masks p. 10

Table S4. Summary of mean RTs and error rates in color-word Stroop task p. 10

Table S5. Relations between frontocortical activity and positive emotional experiences in

the presence and absence of relevant daily events, controlling for age, sex, and frequency of

exposure to positive events p. 11

Table S6. Relations between bilateral dlPFC activity and positive and negative emotional

experiences in the presence and absence of relevant daily events p. 11

Table S7. Relations between MCC activity and positive and negative emotional experiences

in the presence and absence of relevant daily events p. 11

Unilateral effects of the dlPFC on positive and negative emotional experiences in the

presence and absence of relevant daily events p. 12

Table S8. Relations between left dlPFC activity and positive and negative emotional

experiences in the presence and absence of relevant daily events p. 12

Table S9. Relations between right dlPFC activity and positive and negative emotional

experiences in the presence and absence of relevant daily events p. 12

**Supplementary Method**

**Additional information about the current study**

As part of an ongoing prospective longitudinal study examining the etiological mechanisms underlying risk for depression and anxiety disorders, we screened 3281 young adults (75.74% female; *M* = 22 years, *SD* = 2.03) using measures of neuroticism.

During the screening process, participants answered ‘yes’ or ‘no’ for whether they had a history of epilepsy or other neurological disorders, major depressive disorder, bipolar disorder, schizophrenia or other psychotic disorders, substance abuse, were currently taking antipsychotic drugs, or currently undergoing psychotherapy. Participants also completed the Mini International Neuropsychiatric Interview (MINI) (Sheehan et al., 1998) modules for Major Depressive Disorder, General Anxiety Disorder, Panic Disorder, and Social Anxiety Disorder. Participants were additionally screened for MRI contraindications by answering ‘yes’ or ‘no’ for whether they had previously experienced an MRI scan, had gone through a surgery, had metal implants in their body, had extensive tattoos or permanent makeup, were pregnant, had recently experienced dizziness or loss of consciousness, were claustrophobic, and whether they experienced breathing or motor difficulties.

The screening data were stratified into top, middle and bottom quartiles based on the measures of neuroticism. Individuals who met the preliminary inclusion criteria were independently and randomly recruited from each of these three strata. Given the focus of the larger study, we slightly oversampled at-risk individuals in the top quartile (40% top, 30% middle, 30% bottom), thus ensuring a broad range of risk levels in our sample for the development of depression and anxiety disorders.

**Measurement of trait negative emotionality**

Prior to the scanning process, subjects re-completed a set of questionnaires to measure neuroticism: the neuroticism subscales from the NEO five-factor inventory (12 items, Costa & McCrae, 1992, 2008) and the Big Five Inventory (12 items, Soto & John, 2017) and the Behavioral Inhibition Scale (7 items, Gray, 1981). The composite neuroticism score was quantified by averaging the standardized scores of each neuroticism subscale. The composite scores covered a wide range of neuroticism (*z* = −1.89 ~ 1.90).

**
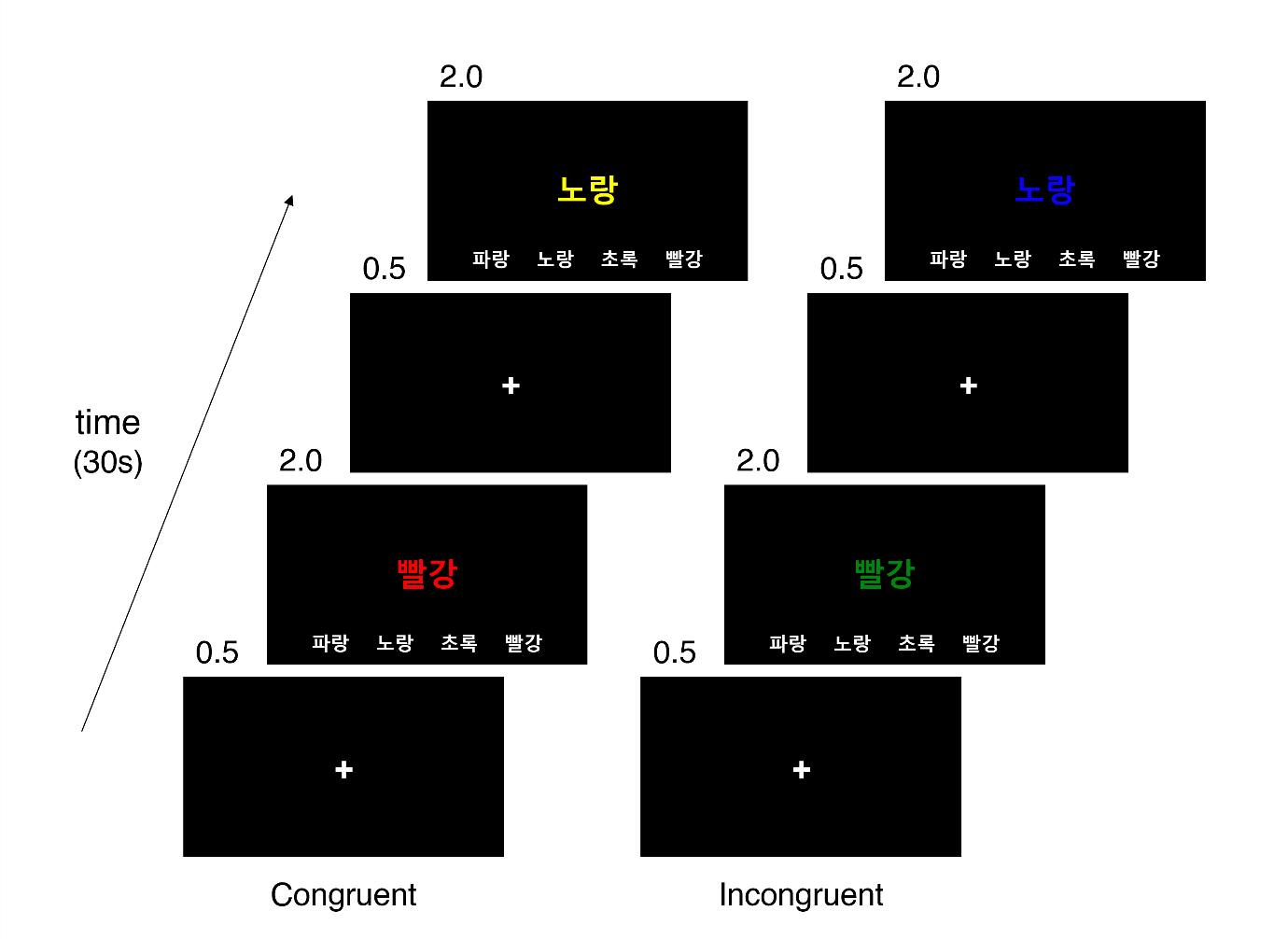
**

**Fig. S1. Color-word Stroop paradigm** (*stimuli presented in Korean*). Example trials illustrate the Stroop word “yellow” (top) and “red” (bottom), each shown in Korean.

**MRI data processing**

***Preprocessing of B0 inhomogeneity mappings.*** A B0-nonuniformity map (or fieldmap) was estimated based on two (or more) echo-planar imaging (EPI) references with *topup* (Jenkinson et al., 2012). In cases where distortion correction resulted in an error, the B0-nonuniformity map was estimated from the phase-drift map(s) measure with two consecutive GRE (gradient-recalled echo) acquisitions. The corresponding phase-map(s) were phase-unwrapped with prelude (FSL 6.0.5.1:57b01774).

***Anatomical data***. The T1-weighted (T1w) image was corrected for intensity non-uniformity (INU) with *N4BiasFieldCorrection* (Tustison et al., 2010), distributed with *ANTs 2.3.3* (Avants et al., 2011, RRID:SCR_004757), and used as T1w-reference throughout the workflow. The T1w-reference was then skull-stripped with a *Nipype* implementation of the *antsBrainExtraction.sh* workflow (from ANTs), using OASIS30ANTs as target template. Brain tissue segmentation of cerebrospinal fluid (CSF), white-matter (WM) and gray-matter (GM) was performed on the brain-extracted T1w using *fast* (FSL 6.0.5.1:57b01774, RRID:SCR_002823, Zhang et al., 2001). Brain surfaces were reconstructed using *recon-all* (FreeSurfer 7.2.0, RRID:SCR_001847, Dale et al., 1999), and the previously estimated brain mask was refined with a custom variation of the method to reconcile ANTs-derived and FreeSurfer-derived segmentations of the cortical gray-matter of Mindboggle (RRID:SCR_002438, Klein et al., 2017). Volume-based spatial normalization to the standard space (MNI152NLin6Asym) was performed through nonlinear registration with *antsRegistration* (ANTs 2.3.3), using brain-extracted versions of both the T1w reference and the T1w template. The following templates were selected for spatial normalization: *FSL’s MNI ICBM 152 non-linear 6th Generation Asymmetric Average Brain Stereotaxic Registration Model* (RRID:SCR_002823, TemplateFlow ID: MNI152NLin6Asym, Evans et al., 2012).

**Supplementary Results**

**Table S1. Results of the exploratory factor analysis on EMA affect items.** EFA yielded a two-factor solution at the between-person level, with factor 1 consisting of all six PA items, and factor 2 consisting of all ten NA items

|  | ML1 | ML2 | Category |
| --- | --- | --- | --- |
| Enthusiastic | **0.61** | 0.28 | PA |
| Joyful | **0.74** | 0.05 |  |
| Cheerful | **0.75** | 0.08 |  |
| Calm | **0.96** | −0.05 |  |
| Content | **0.91** | −0.07 |  |
| Relaxed | **0.96** | −0.05 |  |
| Nervous | −0.06 | **0.85** | NA |
| Worried | −0.07 | **0.83** |  |
| Afraid | 0.03 | **0.92** |  |
| Sad | 0.02 | **0.79** |  |
| Hopeless | 0.08 | **0.82** |  |
| Downhearted | −0.08 | **0.74** |  |
| Irritated | −0.01 | **0.88** |  |
| Angry | −0.24 | **0.51** |  |
| Tired | 0.05 | **0.93** |  |
| Lonely | −0.06 | **0.54** |  |

**
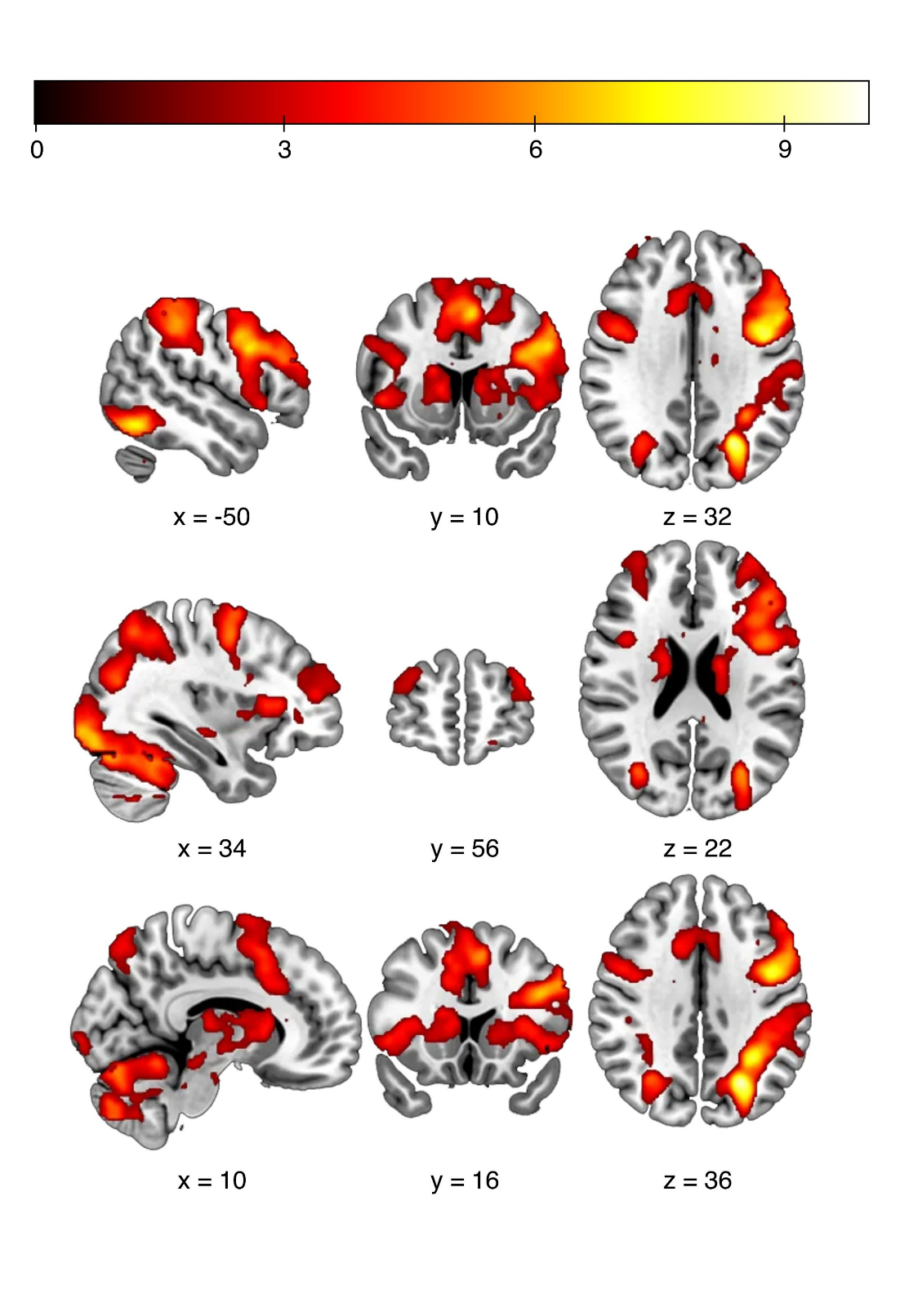
**

**Fig. S2. fMRI task effects.** Consistent with prior research (Huang et al., 2020; Leung et al., 2000; Song & Hakoda, 2015), we observed significant activation in key frontocortical regions, such as the dlPFC and cingulate cortex, during the incongruent condition compared to the congruent condition. (FDR *q* < .05, whole-brain corrected)

**Table 2.** **Descriptive statistics for clusters showing significant activation in the Incongruent > Congruent contrast (FDR *q* < .05, whole-brain corrected)**

|  | **mm^3^** | ***t*** | ***x*** | ***y*** | ***z*** |
| --- | --- | --- | --- | --- | --- |
| Cluster 1 | 311328 |  |  |  |  |
| L Frontal Pole |  | 3.91 | -34 | 56 | 24 |
| L Cingulate Gyrus, anterior division |  | 2.89 | -10 | 22 | 26 |
| L Insular Cortex |  | 4.37 | -36 | 16 | 4 |
| L Frontal Operculum Cortex |  | 4.2 | -46 | 14 | -2 |
| R Right Putamen |  | 3.93 | 20 | 14 | 2 |
| R Frontal Operculum Cortex |  | 5.14 | 48 | 12 | 0 |
| R Right Caudate |  | 4.79 | 12 | 12 | 6 |
| L Paracingulate Gyrus |  | 7.99 | -6 | 10 | 52 |
| R Paracingulate Gyrus |  | 6.01 | 6 | 10 | 54 |
| R Superior Frontal Gyrus |  | 3.47 | 14 | 6 | 70 |
| L Juxtapositional Lobule Cortex (formerly Supplementary Motor Cortex) |  | 7.06 | -2 | 4 | 56 |
| L Precentral Gyrus |  | 9.96 | -42 | 0 | 32 |
| L Left Pallidum |  | 4.68 | -18 | 0 | 6 |
| R Middle Frontal Gyrus |  | 5.62 | 32 | 0 | 62 |
| R Precentral Gyrus |  | 4.75 | 40 | 0 | 32 |
| L Middle Frontal Gyrus |  | 8.55 | -32 | -2 | 54 |
| R Right Pallidum |  | 5.04 | 18 | -4 | 4 |
| L Left Caudate |  | 5.34 | -16 | -6 | 20 |
| R Right Thalamus |  | 5.03 | 16 | -10 | 16 |
| L Left Putamen |  | 3.64 | -30 | -12 | -8 |
| L Left Thalamus |  | 5.97 | -10 | -18 | 10 |
| R Brain-Stem |  | 4.11 | 6 | -18 | -20 |
| L Brain-Stem |  | 4.11 | -10 | -26 | -16 |
| L Supramarginal Gyrus, anterior division |  | 6.79 | -48 | -36 | 46 |
| L Postcentral Gyrus |  | 4.68 | -48 | -36 | 58 |
| R Temporal Fusiform Cortex, posterior division |  | 11.19 | 38 | -38 | -24 |
| L Supramarginal Gyrus, posterior division |  | 4.4 | -44 | -42 | 46 |
| R Superior Parietal Lobule |  | 8.95 | 34 | -48 | 50 |
| L Superior Parietal Lobule |  | 7.21 | -30 | -54 | 46 |
| R Inferior Temporal Gyrus, temporooccipital part |  | 6.55 | 52 | -56 | -16 |
| L Inferior Temporal Gyrus, temporooccipital part |  | 5.32 | -50 | -58 | -14 |
| L Temporal Occipital Fusiform Cortex |  | 11.23 | -36 | -60 | -14 |
| R Lateral Occipital Cortex, superior division |  | 4.04 | 30 | -60 | 50 |
| R Temporal Occipital Fusiform Cortex |  | 7.42 | 44 | -60 | -20 |
| L Lateral Occipital Cortex, superior division |  | 2.68 | -28 | -64 | 46 |
| R Precuneus Cortex |  | 6.86 | 4 | -70 | 54 |
| L Lateral Occipital Cortex, inferior division |  | 2.59 | -46 | -74 | -16 |
| L Lingual Gyrus |  | 7.4 | -2 | -78 | -10 |
| R Occipital Fusiform Gyrus |  | 7.5 | 32 | -80 | -18 |
| R Lingual Gyrus |  | 5.67 | 4 | -80 | -12 |
| L Occipital Fusiform Gyrus |  | 6.37 | -28 | -86 | -16 |
| R Lateral Occipital Cortex, inferior division |  | 7.5 | 38 | -88 | -6 |
| L Occipital Pole |  | 5.67 | -34 | -96 | -6 |
| R Occipital Pole |  | 6.37 | 20 | -96 | -4 |
| Cluster 2 | 2808 |  |  |  |  |
| R Frontal Pole |  | 3.83 | 34 | 48 | 16 |
| Cluster 3 | 2056 |  |  |  |  |
| L Cingulate Gyrus, posterior division |  | 4.15 | -2 | -28 | 26 |
| Cluster 4 | 800 |  |  |  |  |
| R Supramarginal Gyrus, anterior division |  | 3.08 | 46 | -32 | 44 |
| Cluster 5 | 384 |  |  |  |  |
| R Right Thalamus |  | 2.49 | 20 | -30 | -2 |
| Cluster 6 | 296 |  |  |  |  |
| R Brain-Stem |  | 3.51 | 2 | -36 | -44 |
| Cluster 7 | 256 |  |  |  |  |
| L Frontal Pole |  | 3.6 | -24 | 40 | -18 |
| L Frontal Orbital Cortex |  | 2.98 | -22 | 34 | -20 |
| Cluster 8 | 176 |  |  |  |  |
| R Frontal Pole |  | 3.39 | 28 | 42 | -16 |
| Cluster 9 | 152 |  |  |  |  |
| R Brain-Stem |  | 2.91 | 8 | -34 | -26 |
| Cluster 10 | 152 |  |  |  |  |
| R Supramarginal Gyrus, posterior division | 96 | 2.68 | 62 | -38 | 28 |

Note: Although labeled as the precentral gyrus, this area extends into frontolateral regions (Laird et al., 2005) and is anatomically adjacent to the left dlPFC reported in prior studies (e.g. Huang et al., 2020; Leung et al., 2000).

| Region | *t* | *x* | *y* | *z* |
| --- | --- | --- | --- | --- |
| L Dorsolateral Prefrontal Cortex | 8.33 | −50 | 10 | 32 |
| R Dorsolateral Prefrontal Cortex | 3.33 | 34 | 56 | 22 |
| Mid-cingulate Cortex | 3.99 | 10 | 16 | 36 |

**Table S3. Descriptive statistics for local maxima within the bilateral dlPFC and MCC masks**

**Table S4. Summary of mean RTs and error rates in color-word Stroop task**

| Condition | Response time (ms) | Error rates (%) |
| --- | --- | --- |
| Overall | 722.83 (101.91) | 2.87 (2.99) |
| Congruent | 665.22 (90.56) | 1.96 (2.45) |
| Incongruent | 780.44 (118.72) | 3.79 (3.20) |
| Stroop interference | 115.22 (55.16) | 1.83 (0.03) |

Note: This table demonstrates the mean and standard deviation of response time and error rates for the overall task, congruent trials, incongruent trials, and Stroop interference.

**Table S5. Relations between frontocortical activity and positive emotional experiences in the presence and absence of relevant daily events, controlling for age, sex, and frequency of exposure to positive events**

|  | *t* | *β* |
| --- | --- | --- |
| Frontocortical | 0.86 | 0.15 |
| Positive event (vs. Absent) | 19.53*** | 0.59 |
| Age | 1.86 | 0.05 |
| Sex | 2.70** | 0.40 |
| Positive event frequency | 1.47 | 0.46 |
| Frontocortical × Positive event | −2.96** | −0.24 |

*: *p* < .05, **: *p* < .01, ***: *p* < .001

**Table S6. Relations between bilateral dlPFC activity and positive and negative emotional experiences in the presence and absence of relevant daily events**

|  | Positive Affect | | |  | Negative Affect | | |
| --- | --- | --- | --- | --- | --- | --- | --- |
| Factor | *t* | *β* | *SE* | Factor | *t* | *β* | *SE* |
| Bilateral dlPFC | 0.55 | 0.08 | 0.15 | Bilateral dlPFC | 0.04 | 0.004 | 0.10 |
| Positive event  (vs. absent) | 19.85*** | 0.60 | 0.03 | Negative event  (vs. absent) | 11.89*** | 0.48 | 0.04 |
| Bilateral dlPFC  x Positive event | -2.60* | -0.18 | 0.07 | Bilateral dlPFC  x Negative event | -0.87 | -0.08 | 0.09 |

*: *p* < .05, **: *p* < .01, ***: *p* < .001

**Table S7. Relations between MCC activity and positive and negative emotional experiences in the presence and absence of relevant daily events**

|  | Positive Affect | | |  | Negative Affect | | |
| --- | --- | --- | --- | --- | --- | --- | --- |
| Factor | *t* | *β* | *SE* | Factor | *t* | *β* | *SE* |
| MCC | 1.19 | 0.24 | 0.20 | MCC | -0.54 | -0.07 | 0.14 |
| Positive event  (vs. absent) | 19.79*** | 0.60 | 0.03 | Negative event  (vs. absent) | 11.87*** | 0.49 | 0.04 |
| MCC  x Positive event | -2.90** | -0.27 | 0.09 | MCC  x Negative event | 0.45 | 0.05 | 0.12 |

*: *p* < .05, **: *p* < .01, ***: *p* < .001

**Unilateral effects of the dlPFC on positive and negative emotional experiences in the presence and absence of relevant daily events**

For exploratory purposes, we additionally tested unilateral effects of the dlPFC on positive and negative emotional experience in the presence and absence of relevant events. Both left and right dlPFC activity were found to modulate positive emotional experience in the absence of positive events, consistent with the main findings (*ps* < .05; see Supplementary Table S8 and S9); however, the effect of the right dlPFC did not survive correction for multiple comparisons. Neither left nor right dlPFC activity was associated with negative emotional experience in the presence or absence of negative events (*ps* > .41; see Supplementary Table S8 and S9).

**Table S8.** **Relations between left dlPFC activity and positive and negative emotional experiences in the presence and absence of relevant daily events**

|  | Positive Affect | | |  | Negative Affect | | |
| --- | --- | --- | --- | --- | --- | --- | --- |
| Factor | *t* | *β* | *SE* | Factor | *t* | *β* | *SE* |
| Left dlPFC | 0.88 | 0.11 | 0.13 | Left dlPFC | -0.75 | -0.07 | 0.87 |
| Positive event  (vs. absent) | 19.88*** | 0.60 | 0.03 | Negative event  (vs. absent) | 11.90*** | 0.48 | 0.04 |
| Left dlPFC  x Positive event | -2.55* | -0.15 | 0.06 | Left dlPFC  x Negative event | -0.82 | -0.06 | 0.07 |

*: *p* < .05, **: *p* < .01, ***: *p* < .001

**Table S9. Relations between right dlPFC activity and positive and negative emotional experiences in the presence and absence of relevant daily events**

|  | Positive Affect | | |  | Negative Affect | | |
| --- | --- | --- | --- | --- | --- | --- | --- |
| Factor | *t* | *β* | *SE* | Factor | *t* | *β* | *SE* |
| Right dlPFC | 0.003 | 0.0004 | 0.15 | Right dlPFC | 1.05 | 0.10 | 0.10 |
| Positive event  (vs. absent) | 19.40*** | 0.59 | 0.03 | Negative event  (vs. absent) | 11.86*** | 0.48 | 0.04 |
| Right dlPFC  x Positive event | -2.04* | -0.14 | 0.07 | Right dlPFC  x Negative event | -0.73 | -0.06 | 0.08 |

*: *p* < .05, **: *p* < .01, ***: *p* < .001

**References**

Avants, B. B., Tustison, N. J., Song, G., Cook, P. A., Klein, A., & Gee, J. C. (2011). A reproducible evaluation of ANTs similarity metric performance in brain image registration. *Neuroimage*, *54*(3), 2033-2044. <https://doi.org/10.1016/j.neuroimage.2010.09.025>

Costa, P. T., & McCrae, R. R. (1992). Normal personality assessment in clinical practice: The NEO Personality Inventory. *Psychological assessment*, *4*(1), 5. <https://doi.org/10.1037/1040-3590.4.1.5>

Costa, P. T., & McCrae, R. R. (2008). The revised neo personality inventory (neo-pi-r). *The SAGE handbook of personality theory and assessment*, *2*(2), 179-198.

Dale, A. M., Fischl, B., & Sereno, M. I. (1999). Cortical Surface-Based Analysis: I. Segmentation and Surface Reconstruction. *Neuroimage*, *9*(2), 179-194. <https://doi.org/10.1006/nimg.1998.0395>

Evans, A. C., Janke, A. L., Collins, D. L., & Baillet, S. (2012). Brain templates and atlases. *Neuroimage*, *62*(2), 911-922. <https://doi.org/10.1016/j.neuroimage.2012.01.024>

Gray, J. A. (1981). A critique of Eysenck’s theory of personality. In *A model for personality* (pp. 246-276). Springer. <https://doi.org/10.1007/978-3-642-67783-0_8>

Huang, Y., Su, L., & Ma, Q. (2020). The Stroop effect: An activation likelihood estimation meta-analysis in healthy young adults. *Neuroscience letters*, *716*, 134683. <https://doi.org/10.1016/j.neulet.2019.134683>

Jenkinson, M., Beckmann, C. F., Behrens, T. E., Woolrich, M. W., & Smith, S. M. (2012). Fsl. *Neuroimage*, *62*(2), 782-790. <https://doi.org/10.1016/j.neuroimage.2011.09.015>

Klein, A., Ghosh, S. S., Bao, F. S., Giard, J., Häme, Y., Stavsky, E., Lee, N., Rossa, B., Reuter, M., & Chaibub Neto, E. (2017). Mindboggling morphometry of human brains. *PLoS computational biology*, *13*(2), e1005350. <https://doi.org/10.1371/journal.pcbi.1005350>

Laird, A. R., McMillan, K. M., Lancaster, J. L., Kochunov, P., Turkeltaub, P. E., Pardo, J. V., & Fox, P. T. (2005). A comparison of label‐based review and ALE meta‐analysis in the Stroop task. *Human brain mapping*, *25*(1), 6-21. <https://doi.org/10.1002/hbm.20129>

Leung, H.-C., Skudlarski, P., Gatenby, J. C., Peterson, B. S., & Gore, J. C. (2000). An event-related functional MRI study of the Stroop color word interference task. *Cerebral cortex*, *10*(6), 552-560. <https://doi.org/10.1093/cercor/10.6.552>

Sheehan, D. V., Lecrubier, Y., Sheehan, K. H., Amorim, P., Janavs, J., Weiller, E., Hergueta, T., Baker, R., & Dunbar, G. C. (1998). The Mini-International Neuropsychiatric Interview (MINI): the development and validation of a structured diagnostic psychiatric interview for DSM-IV and ICD-10. *J clin psychiatry*, *59*(Suppl 20), 22-33.

Song, Y., & Hakoda, Y. (2015). An fMRI study of the functional mechanisms of Stroop/reverse-Stroop effects. *Behavioural brain research*, *290*, 187-196. <https://doi.org/10.1016/j.bbr.2015.04.047>

Soto, C. J., & John, O. P. (2017). The next Big Five Inventory (BFI-2): Developing and assessing a hierarchical model with 15 facets to enhance bandwidth, fidelity, and predictive power. *Journal of personality and social psychology*, *113*(1), 117. <https://doi.org/10.1037/pspp0000096>

Tustison, N. J., Avants, B. B., Cook, P. A., Zheng, Y., Egan, A., Yushkevich, P. A., & Gee, J. C. (2010). N4ITK: Improved N3 Bias Correction. *IEEE Transactions on Medical Imaging*, *29*(6), 1310-1320. <https://doi.org/10.1109/TMI.2010.2046908>

Zhang, Y., Brady, M., & Smith, S. (2001). Segmentation of brain MR images through a hidden Markov random field model and the expectation-maximization algorithm. *IEEE Transactions on Medical Imaging*, *20*(1), 45-57. <https://doi.org/10.1109/42.906424>
